# Effect of Topical Vancomycin Powder on Surgical Site Infections in Cranioplasty: A Systematic Review and Meta-Analysis

**DOI:** 10.7759/cureus.91183

**Published:** 2025-08-28

**Authors:** Amr El Mohamad, Sadeen Eid, Ali Msheik, Omar Shihadeh, Abdallah Basit W Nuru-Ahmed, Mohamad El Mohamad, Alaaeldin Ali Salih Ahmed, Ibrahim Abdulhafeez, Arshad Ali

**Affiliations:** 1 Neurosurgery, Hamad General Hospital, Doha, QAT; 2 Medical School, Jordan University of Science and Technology, Amman, JOR; 3 Neurosurgery, Hamad Medical Corporation, Doha, QAT; 4 General Practice, Effia Nkwanta Regional Hospital, Takoradi, GHA; 5 General Practice, Biruni University, Istanbul, TUR; 6 Neurological Surgery, Hamad General Hospital, Doha, QAT; 7 Clinical Academic Sciences, Qatar University, Doha, QAT; 8 Neurological Sciences, Weill Cornell Medicine, Ar-Rayyan, QAT

**Keywords:** anti-bacterial agents/therapeutic use, cranioplasty, meta-analysis, surgical wound infection control, surgical wound infection prevention, systematic reviews, vancomycin administration and dosage, vancomycin powder

## Abstract

Surgical site infections remain a significant complication after cranioplasty, increasing morbidity, hospitalization, and the need for reoperation. While intrawound vancomycin powder has shown efficacy in reducing surgical site infections in other neurosurgical procedures, its role in cranioplasty remains unclear. This systematic review and meta-analysis evaluated the effectiveness of vancomycin powder in preventing surgical site infections after cranioplasty. A comprehensive search of PubMed, Cochrane Library, and Embase was conducted through April 2025. Eligible studies included randomized controlled trials and observational studies comparing vancomycin powder versus no vancomycin powder in cranioplasty, reporting surgical site infection outcomes. Risk of bias was assessed. Pooled odds ratios with 95% confidence intervals were calculated using a random-effects model. Heterogeneity was assessed with I² statistics. Three retrospective observational studies were included. The results showed a non-significant trend toward reduced infection with vancomycin powder. Heterogeneity was moderate. Studies varied in vancomycin powder dosing, timing, implant materials, and surgical techniques. Overall risk of bias was moderate, mainly due to missing data. No vancomycin powder-related adverse events were reported. Current evidence suggests a non-significant trend toward surgical site infection reduction with intrawound vancomycin powder in cranioplasty. However, limited sample size, study heterogeneity, and retrospective designs preclude firm conclusions. High-quality randomized trials are needed to establish vancomycin powder’s efficacy and safety in this context.

## Introduction and background

Decompressive craniectomy is a neurosurgical procedure frequently performed to decrease elevated intracranial pressure following traumatic brain injury, stroke, or other conditions causing significant cerebral edema [1]. The resulting cranial defect can lead to a variety of complications, including neurological deficits, cosmetic disfigurement, and an increased risk of injury to the underlying brain [2]. Cranioplasty, the surgical repair of the cranial defect, is performed to restore skull integrity, protect the brain, and improve neurological function. The timing of cranioplasty can vary, with some studies suggesting earlier reconstruction may lead to improved outcomes [3,4]. Restoring the cranial defect through cranioplasty can lead to neuropsychological improvements in both motor and cognitive skills, serving as a reminder to rehabilitation clinicians to give serious consideration to prompt performance of cranioplasty during rehabilitation [5,6]. Cranioplasty is associated with a significant risk. The overall complication rate from cranioplasty is around 30% [7], and surgical site infection (SSI) rates range from 3.7% to 25.6%, which can lead to increased morbidity, prolonged hospital stays, additional surgical procedures, long-term antibiotic therapy, and higher healthcare costs [1]. Multiple approaches have been explored to reduce the risk of infection in cranioplasty surgery, including the application of antibiotics directly at the surgical site. Vancomycin powder has been used in spine surgery and has shown good results [8]. It gained popularity in non-spinal neurosurgery, with a meta-analysis showing an overall beneficial effect on SSIs [2]. Another study also revealed the potential benefit of vancomycin in the craniotomy procedure [9]. The use of vancomycin powder in cranioplasty has yielded inconsistent results in various studies [1,10], necessitating a meta-analysis to comprehensively assess its efficacy. In this study, we conducted a systematic review and meta-analysis to evaluate the efficacy of vancomycin powder in reducing the risk of SSIs in cranioplasty procedures.

## Review

Methodology

This systematic review and meta-analysis was registered on the International Prospective Register of Systematic Reviews (Prospero) under the protocol number CRD420251038985 [11]. This study was conducted according to the Cochrane Handbook for Systematic Reviews of Interventions [12] and reported according to the Preferred Reporting Items for Systematic Reviews Incorporating Network Meta-analyses (PRISMA-NMA) guidelines [13].

Eligibility Criteria

We included randomized controlled trials and observational studies that enrolled patients undergoing cranioplasty and compared the use of local vancomycin powder with no vancomycin. Studies were eligible if they reported SSI as an outcome. Given that the diagnostic criteria for SSI were not consistently defined across all studies, we accepted the definition provided by each study, acknowledging this variability as a potential source of outcome ascertainment bias. We excluded studies involving patients who underwent craniotomy or other non-cranioplasty neurosurgical procedures, as well as studies that did not report the outcome of interest. No restrictions were applied regarding the date or language of publication.

Search Strategy and Data Extraction

We searched PubMed, Cochrane, and Embase from inception to April 2025 using the following search strategy: (cranioplasty AND vancomycin) OR (“intrawound vancomycin” AND cranioplasty) OR (“topical vancomycin” AND cranioplasty). The same retrieval strategy was also applied to all the databases, tailoring the research strings to accommodate the recommendations of each database using research strings included in the Appendix. We also manually searched the references from all included studies, previous systematic reviews, and meta-analyses for any additional studies. Two authors (AEM and SE) independently selected the studies and extracted the data according to the inclusion and exclusion criteria. A third author (AM) settled any disagreement between the two authors.

Quality Assessment

Risk of bias was assessed using the Risk of Bias in Non-randomized Studies of Interventions (ROBINS-I) tool. This framework evaluates bias across the following seven domains: confounding, selection of participants, classification of interventions, deviations from intended interventions, missing data, measurement of outcomes, and selection of reported results. Each study was independently reviewed by two authors, with disagreements resolved by consensus.

Statistical Analysis

Statistical analysis was performed using Review Manager (RevMan) version 5.4. Pooled odds ratios (ORs) with 95% confidence intervals (CIs) were calculated for dichotomous outcomes using a random-effects model (Mantel-Haenszel method). Heterogeneity was assessed using the chi-square test and quantified with the I² statistic and tau-squared (τ²). Potential sources of heterogeneity included differences in vancomycin powder dosing, variations in application technique (placement directly on bone flap versus dural surface), and variability in SSI definitions across studies. A funnel plot was used to visually assess potential publication bias. Due to the limited number of studies (n = 3), formal tests for asymmetry were not conducted, and findings were interpreted with caution. A sensitivity analysis excluding the study with the most divergent effect size was performed, which did not materially alter the direction or significance of the pooled estimate. A p-value <0.05 was considered statistically significant.

Results

**Figure 1:**
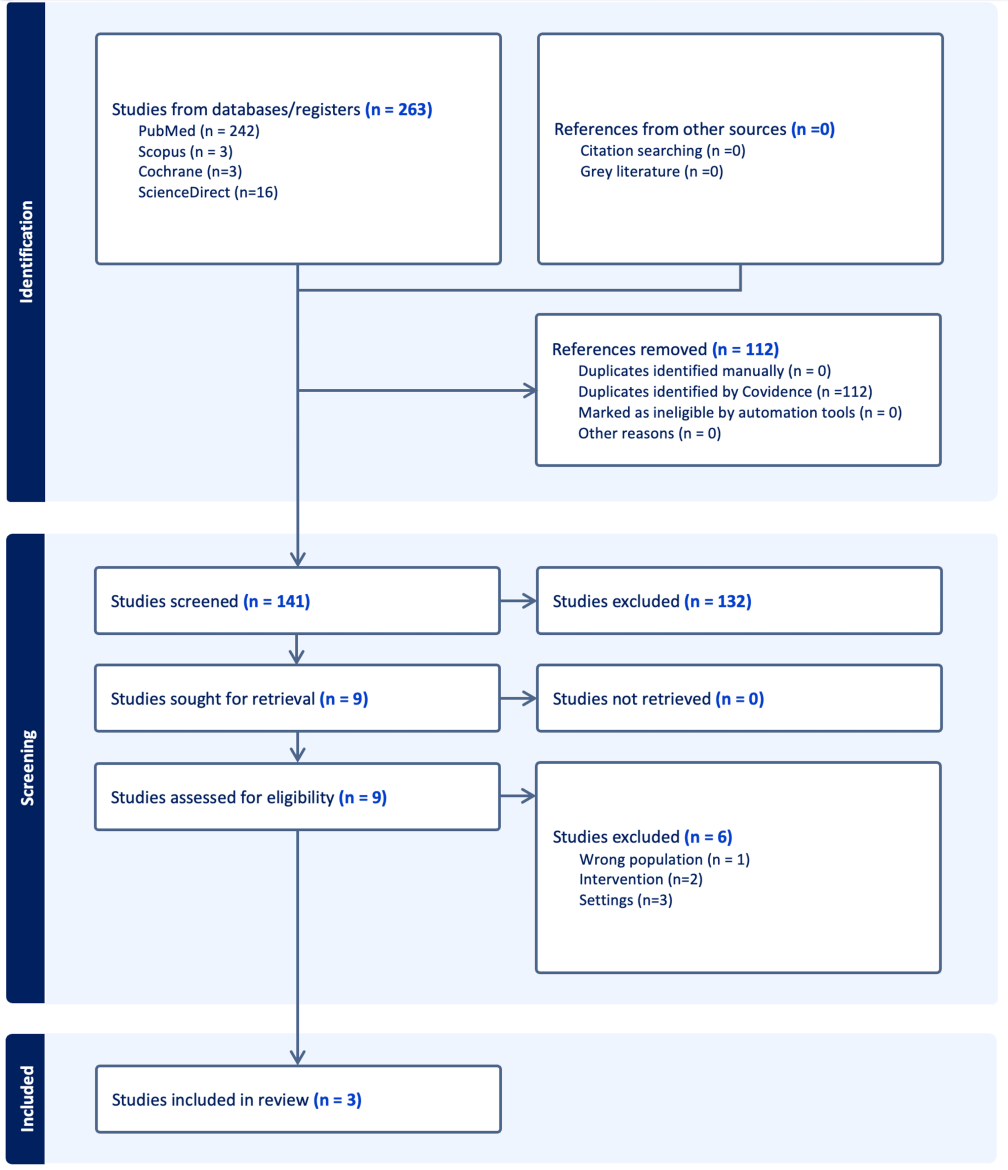
Preferred Reporting Items for Systematic Reviews Incorporating Network Meta-analyses flow diagram showing database searches, screening, and selection of studies.

Our systematic search yielded 263 potential articles, as shown in Figure 1. After removing duplicates, 141 studies were reviewed based on the title and abstract, and 132 records were excluded. Nine studies were thoroughly reviewed for inclusion and exclusion criteria. Ultimately, three observational studies were included, investigating the impact of intrawound vancomycin powder on SSI following cranioplasty [1,10,14]. The characteristics of the study populations and relevant surgical variables are summarized in Table 1.

**Table 1:** Summary of included retrospective studies comparing SSI rates between VP and control groups in cranioplasty patients. SSI: surgical site infection; BMI: body mass index; DM: diabetes mellitus; HTN: hypertension; prosthetic implant: acrylic or titanium; VP: vancomycin powder cohort

Characteristic	Abode-Iyamah et al., 2018 (VP) [1]	Abode-Iyamah et al., 2018 (Control) [1]	Caruso et al., 2021 (VP) [14]	Caruso et al., 2021 (Control) [14]	Youn et al., 2023 (VP) [10]	Youn et al., 2023 (Control) [10]
Number of patients	92	166	19	52	105	475
Study design	Retrospective	Retrospective	Retrospective	Retrospective	Retrospective	Retrospective
Center	University of Iowa Hospitals	Univ. of Iowa Hospitals	-	-	Bundang Jesaeng General Hospital	Bundang Jesaeng General Hospital
Year of recruitment	2008–2014	2008–2014	2014–2018	2014–2018	1998–2021	1998–2021
Follow-up (months)	3	3	12	12	12	12
Number of SSI patients	6 (6.5%)	9 (5.4%)	1 (5.2%)	2 (3.8%)	0	31 (6.5%)
Mean age (years)	49.3	47.5	-	-	52	53
Male sex	59 (64.1%)	100 (60.2%)	-	-	57 (54.3%)	272 (57.3%)
Median BMI (kg/m²)	26.7	36.2	-	-	22.5	22.7
DM	13 (14.1%)	13 (7.8%)	-	-	17 (16.2%)	35 (7.4%)
HTN	-	-	-	-	42 (40%)	161 (33.9%)
Smoking	43 (46.7%)	75 (45.2%)	-	-	24 (22.9%)	168 (35.4%)
Indication: hemorrhage	41 (44.6%)	58 (34.9%)	-	-	54 (51.4%)	252 (53%)
Indication: trauma	48 (52.2%)	91 (54.8%)	-	-	34 (32.4%)	163 (34.3%)
Indication: tumor	2 (2.2%)	19 (11.1%)	-	-	4 (3.8%)	27 (5.7%)
Indication: other	1 (1.1%)	1 (0.6%)	-	-	13 (12.4%)	33 (7%)
Mean operation duration (minutes)	137.9	143.8	-	-	119	120
Implant: native	52 (56.5%)	103 (62.1%)	-	-	92 (87.6%)	417 (78.8%)
Implant: prosthetic	40 (43.5%)	63 (38%)	-	-	13 (12.4%)	58 (12.2%)

**Figure 2:**
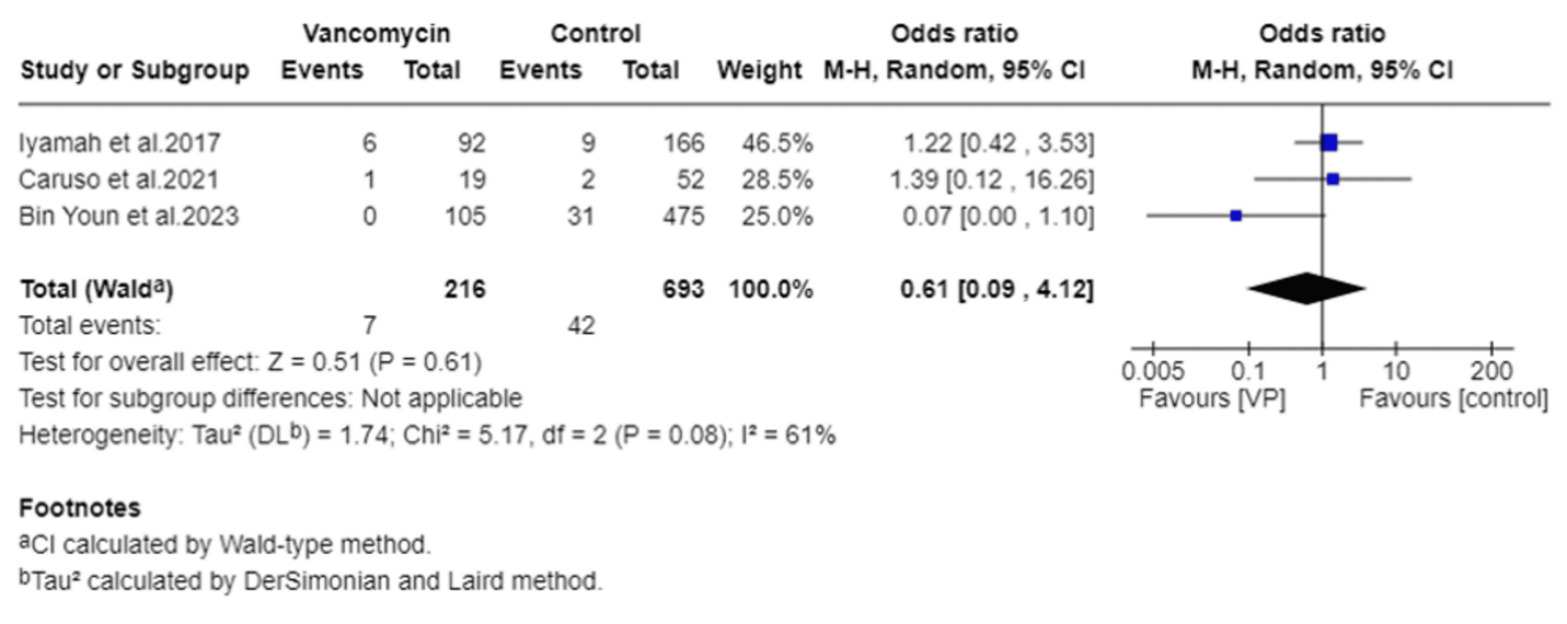
Summary of the data and odds ratio for each study individually and combined results with a forest plot. For each study, the number of patients who had craniotomies with or without topical vancomycin is shown as (total) and the number of patients who had surgery.

A total of 909 patients were analyzed, including 216 in the vancomycin group and 693 in the control group. The pooled OR for SSIs in patients receiving vancomycin powder was 0.61 (95% CI = 0.09 to 4.12), indicating a statistically nonsignificant reduction in infection risk (Z = 0.51, p = 0.61) (Figure 2). The very wide CI and the small number of included retrospective studies suggest that this result should be interpreted with caution.

Effect estimates varied across the included studies. Abode-Iyamah et al. reported an OR of 1.22 (95% CI = 0.42 to 3.53), Caruso et al. reported an OR of 1.39 (95% CI = 0.12 to 16.26), and Youn et al. demonstrated a substantially lower OR of 0.07 (95% CI = 0.00 to 1.10). Moderate heterogeneity was observed among the studies (I² = 61%), with a chi-square value of 5.17 (df = 2, p = 0.08) and tau² of 1.74. Potential sources of heterogeneity included variations in vancomycin powder dosing, timing of administration, and application technique (such as placement directly on the bone flap versus the dural surface), as well as differences in SSI definitions and follow-up durations across studies. A sensitivity analysis was performed, excluding the study with the most divergent effect size. This did not materially change the direction or statistical significance of the pooled results.

**Figure 3:**
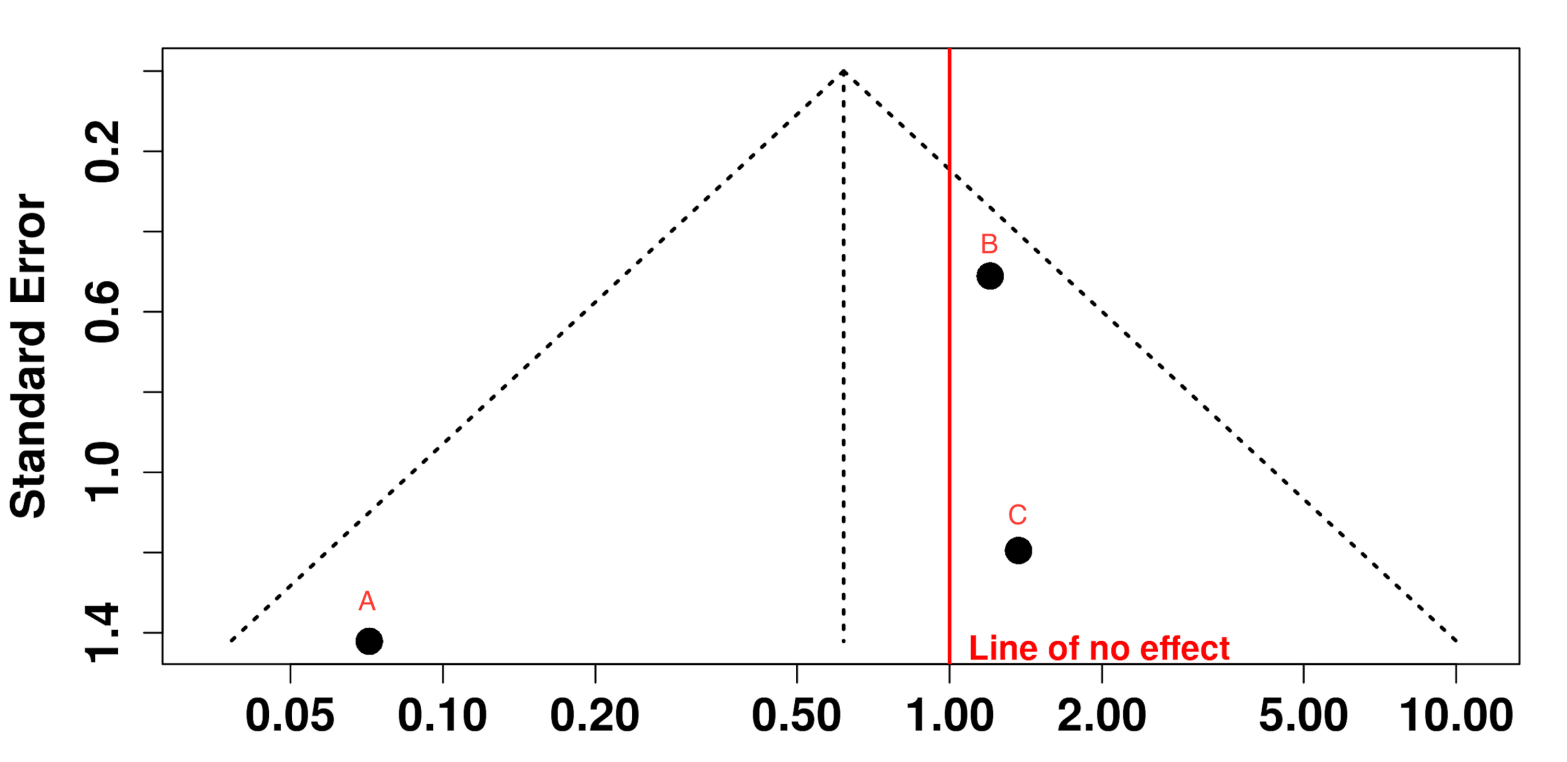
Funnel plot showing distribution of studies according to their sizes and results. The three studies are not distributed symmetrically. Y-axis: standard error (SE); X-axis: odds ratio (OR). A: Youn et al., 2023 [10]; B: Abode-Iyamah et al., 2017 [1]; C: Caruso et al., 2021 [14].

A funnel plot was constructed to assess potential publication bias (Figure 3). The distribution of the three studies was mildly asymmetric, with the study by Youn et al. (2023) exhibiting a lower OR and higher standard error, possibly reflecting a small-study effect. However, the small number of studies (n = 3) limits the interpretability of the funnel plot, and while publication bias cannot be excluded, conclusions remain inconclusive. This limitation was acknowledged, and findings from the visual assessment should be interpreted with caution.

The three included studies were evaluated using the ROBINS-I V2 tool, and the results are summarized in Table 2. Overall, all studies demonstrated low risk of bias across most domains. However, two studies were judged to have a moderate risk of bias in the domain of missing data and confounding factors, as they did not describe strategies for handling incomplete outcome data or loss to follow-up. Despite these limitations, all studies were considered methodologically acceptable and were included in the quantitative synthesis.

**Table 2:** Risk of bias assessment using ROBINS-I V2. ROBINS-I V2: Risk of Bias in Non-randomized Studies of Interventions Version 2

Domain	Caruso et al., 2021 [14]	Abode-Iyamah et al., 2018 [1]	Youn et al., 2023 [10]
Confounding	Moderate	Moderate	Low
Classification of interventions	Low	Low	Low
Selection of participants	Low	Low	Low
Deviations from intended interventions	Low	Low	Low
Missing data	Moderate	Moderate	Low
Measurement of outcomes	Low	Low	Low
Selection of reported result	Low	Low	Low
Overall bias	Moderate	Moderate	Low

Each study was retrospective in design, introducing the possibility of recall bias, as SSI diagnoses were based on surgical records that may vary in completeness. Nonetheless, attrition bias is unlikely, given that postoperative wound assessments were routinely performed during hospitalization and follow-up visits. No loss to follow-up was reported in any of the studies.

Discussion

In this systematic review and meta-analysis of three studies and 909 patients, we assessed the use of vancomycin powder in cranioplasty cases. The main findings indicated a trend toward decreased risk of SSI with the use of vancomycin powder, but the results were not statistically significant, with an OR of 0.61 (95% CI = 0.09 to 4.12). This contrasted with the previous reported findings for other neurosurgical procedures. Bokhari et al. assessed in their meta-analysis the use of vancomycin powder in different non-spinal neurosurgical procedures, including cranioplasty, ventriculo-peritoneal shunt insertion, and deep brain stimulation, suggesting an overall beneficial effect on SSI incidence, although this was not consistently observed across all sub-procedures [2]. Deora et al. conducted an updated meta-analysis, which showed a decreased risk of SSI in all subgroups, including ventriculo-peritoneal shunt insertion, deep brain stimulation, and craniotomy, except for cranioplasty, where there was only one study included [15]. A side-by-side comparison of our findings with those from previous meta-analyses and related neurosurgical studies is presented in Table 3.

**Table 3:** Comparison of the present systematic review and meta-analysis with previous studies on topical VP in neurosurgical procedures. This table contrasts the findings of the present review on VP use in cranioplasty with previous meta-analyses and large observational studies in neurosurgery. Data include study population, surgical procedure type, pooled or individual ORs for SSI, and key methodological notes. The comparison highlights differences in patient cohorts, surgical settings, and VP application protocols, which may explain variations in outcomes. VP: vancomycin powder; SSI: surgical site infection; VPS: ventriculoperitoneal shunt; DBS: deep brain stimulation; OR: odds ratio; CI: confidence interval

Study	Population/Procedure	Number of studies/patients	Effect on SSI (OR, 95% CI)	Subgroup findings	Conclusions
Current review (2025)	Cranioplasty only	3/909	0.61 (0.09–4.12), p = 0.61; NS	No statistically significant benefit in cranioplasty; effect varies by study	Wide CI, moderate heterogeneity; only retrospective studies; cranioplasty-specific
Bokhari et al., 2018 [2]	Mixed non-spinal neurosurgery (including cranioplasty, VPS, DBS)	11/3,284	0.61 (0.44–0.85); significant ↓ SSI overall	Benefit observed in pooled analysis, but inconsistent across sub-procedures	Included mixed neurosurgical procedures; some heterogeneity
Deora et al., 2021 [15]	Mixed non-spinal neurosurgery (including cranioplasty, VPS, DBS, craniotomy)	15/6,944	0.58 (0.45–0.75); significant ↓ SSI overall	Benefit in all subgroups except cranioplasty (only one study included)	Updated review; still limited cranioplasty-specific data

While a previous meta-analysis demonstrated a significant reduction in SSI rates following craniotomy with topical vancomycin use [9], the applicability of these findings to cranioplasty remains limited. Cranioplasty is a delayed, secondary procedure that typically involves reoperation through scarred tissues, often with the implantation of foreign materials [16]. These factors inherently increase the risk of complications compared to primary craniotomy. Moreover, differences in wound environment, tissue viability, and healing dynamics may alter the efficacy of topical antibiotics. Recognizing the need for clarity in surgical outcomes, we conducted this comprehensive meta-analysis to assess the impact of vancomycin powder in the cranioplasty setting. In contrast to prior findings in craniotomy, our review did not demonstrate a statistically significant reduction in SSIs with vancomycin powder use following cranioplasty (pooled OR = 0.61, 95% CI = 0.09 to 4.12; p = 0.61). The CI is very wide and crosses the line of no effect, encompassing the possibility of both meaningful benefit and potential harm, underscoring the high degree of imprecision. These results remain inconclusive and should be interpreted with caution, highlighting the need for well-designed prospective studies with adequate sample sizes and standardized vancomycin powder protocols, focused specifically on the cranioplasty population.

The three included studies demonstrated varying results regarding the efficacy of vancomycin powder in preventing SSIs following cranioplasty. Abode-Iyamah et al. conducted one of the first studies to explore this application, analyzing a retrospective cohort of 258 patients undergoing first-time cranioplasty [1]. No significant reduction in SSIs was observed in the vancomycin powder group compared to controls (6.5% vs. 5.4%, p = 0.72). Notably, this study included a heterogeneous population with both autologous and alloplastic implants. Multivariate analysis identified prior ipsilateral craniotomy and the use of prosthetic materials as independent risk factors for infection.

Caruso et al. retrospectively analyzed 71 patients who underwent autologous cranioplasty exclusively for traumatic brain injury [16]. They reported a low overall SSI rate (4.2%) and no significant difference between the vancomycin powder and control groups (p = 0.99). The limited sample size, homogenous patient population, and exclusive use of autologous bone may have contributed to the lack of observed benefit and reduced statistical power.

In contrast, Youn et al. reported a significant reduction in SSIs within a large retrospective cohort of 580 patients. No infections occurred in the vancomycin powder group (0/105), compared to a 6.5% infection rate in the control group (31/475), yielding a statistically significant difference (p = 0.026) [10]. This study included a broader case mix encompassing traumatic, vascular, and neoplastic etiologies and utilized both autologous and prosthetic implants. A key methodological difference was the standardized vancomycin powder protocol: a fixed dose of 1 g was split between intradural and epidural surfaces before bone flap fixation, unlike the other two studies, where vancomycin powder was applied only after bone placement and in variable doses (500 mg to 2 g).

These discrepancies may be attributed to several sources of clinical and methodological heterogeneity, including differences in patient populations, cranioplasty materials, vancomycin dosing, application techniques, and sample sizes. Collectively, these factors may influence local antibiotic bioavailability and tissue penetration, thus affecting the overall impact of vancomycin powder on postoperative infection risk.

Previous research has investigated the impact of implant material on postoperative infection rates. Abode-Iyamah et al. found that prosthetic implants were associated with a significantly increased risk of SSI compared to autologous bone (OR = 3.93, p < 0.05) [1]. Similarly, Kim et al. reported a high incidence of SSI in prosthetic implants compared to autologous (8.2% vs. 6.7%), although not statistically significant [17]. In contrast, other studies have found no statistically significant difference between synthetic and native material, highlighting an ongoing controversy in this area [7,18]. On the other hand, the indication of decompressive craniectomy may affect the risk of SSI, with hemorrhagic stroke being a predictor of SSI [7]. Although the included studies reported implant type and surgical indication, none provided stratified infection outcomes according to both vancomycin use and subgroup characteristics (implant type or indication). Therefore, it was not possible to assess whether the efficacy of topical vancomycin differs between patients with native versus prosthetic implants or across different surgical indications for decompressive craniectomy. Further studies with individual patient-level data or stratified subgroup reporting are needed to explore potential treatment-subgroup interactions.

The timing of cranioplasty is another critical factor influencing the risk of SSI. Historically, early cranioplasty was thought to be associated with higher rates of SSI and overall complications, leading to recommendations to delay the procedure to avoid intervening in a potentially contaminated field [15,19,20]. However, more recent studies have challenged this view, reporting no significant increase in SSI risk with early cranioplasty, and suggesting a potential advantage in terms of improved functional and psychological recovery through earlier rehabilitation [3,18,20-22]. Despite these findings, the impact of timing on infection rates remains a subject of debate. In our review, we could not evaluate the influence of vancomycin powder on early versus late cranioplasty due to insufficient data in the included studies.

Although several side effects, such as seroma formation, wound dehiscence, ototoxicity, and nephrotoxicity, have been reported in the literature, primarily in the context of spinal surgery where vancomycin powder has been most extensively studied [23], it is generally regarded as a safe and effective measure for reducing postoperative SSIs, with a low associated morbidity. In our review, no direct adverse events attributable to vancomycin were observed.

Limitations

Our study has several limitations. First, only three retrospective studies were included in the meta-analysis, thus limiting the statistical power and the external validity of our findings. Moreover, none of the studies provided stratified SSI outcomes by both vancomycin use and subgroup characteristics such as implant type or surgical indication. As a result, we were unable to conduct subgroup meta-analyses to evaluate potential effect modifiers. Second, all included studies were non-randomized and retrospective, carrying an inherent risk of selection bias and unmeasured confounding. The ROBINS-I tool was used to assess the risk of bias, with all studies demonstrating a low risk of bias in most domains (Table 2). However, two of the studies showed a high risk of bias related to incomplete follow-up, as none reported strategies to address missing data. Finally, moderate heterogeneity was observed among the studies, which may be related to the different surgical techniques. These limitations highlight the need for high-quality randomized clinical trials to assess the effectiveness of vancomycin powder in cranioplasty.

## Conclusions

This systematic review and meta-analysis investigated the use of vancomycin powder in cranioplasty and its effect on SSI. While a trend toward reduced SSIs was observed, there was no statistically significant decrease associated with vancomycin powder use. Given the wide CIs, variability in application protocols, and the small number of retrospective studies, these findings should be considered hypothesis-generating rather than definitive. Although vancomycin powder has shown benefit in other neurosurgical procedures, its role in cranioplasty remains uncertain. High-quality randomized controlled trials with standardized vancomycin powder dosing and application methods are required to clarify its efficacy.

## Appendices

Search strategy

PubMed

(“Cranioplasty”[Mesh] OR cranioplast*[tiab] OR “cranial reconstruct*”[tiab] OR “skull reconstruct*”[tiab] OR “bone flap”[tiab]) AND ((“Vancomycin”[Mesh] OR vancomycin[tiab]) AND (“Administration, Topical”[Mesh] OR topical[tiab] OR intrawound[tiab] OR local[tiab] OR powder[tiab] OR “vancomycin powder”[tiab] OR “topical vancomycin”[tiab] OR “intrawound vancomycin”[tiab])) AND (“Surgical Wound Infection”[Mesh] OR “surgical site infection*”[tiab] OR “wound infection*”[tiab] OR “postoperative infection*”[tiab]) NOT (spine*[tiab] OR spinal[tiab] OR laminect*[tiab] OR scoliosis[tiab])

Embase

(‘cranioplasty’/exp OR cranioplast*:ti,ab OR ‘cranial reconstruct*’:ti,ab OR ‘skull reconstruct*’:ti,ab OR ‘bone flap’:ti,ab) AND ((‘vancomycin’/exp OR vancomycin:ti,ab) AND (‘topical drug administration’/exp OR topical:ti,ab OR intrawound:ti,ab OR local:ti,ab OR powder:ti,ab OR ‘vancomycin powder’:ti,ab OR ‘topical vancomycin’:ti,ab OR ‘intrawound vancomycin’:ti,ab)) AND (‘surgical wound infection’/exp OR ‘surgical site infection*’:ti,ab OR ‘wound infection*’:ti,ab OR ‘postoperative infection*’:ti,ab) NOT (spine*:ti,ab OR spinal:ti,ab OR laminect*:ti,ab OR scoliosis:ti,ab)

Cochrane Library

#1 MeSH descriptor: [Cranioplasty] explode all trees

#2 cranioplast*:ti,ab,kw OR “cranial reconstruct*”:ti,ab,kw OR “skull reconstruct*”:ti,ab,kw OR “bone flap”:ti,ab,kw

#3 #1 OR #2

#4 MeSH descriptor: [Vancomycin] explode all trees

#5 vancomycin:ti,ab,kw

#6 MeSH descriptor: [Administration, Topical] explode all trees

#7 topical:ti,ab,kw OR intrawound:ti,ab,kw OR local:ti,ab,kw OR powder:ti,ab,kw

#8 “vancomycin powder”:ti,ab,kw OR “topical vancomycin”:ti,ab,kw OR “intrawound vancomycin”:ti,ab,kw

#9 (#4 OR #5)

#10 (#6 OR #7 OR #8)

#11 #9 AND #10

#12 MeSH descriptor: [Surgical Wound Infection] explode all trees

#13 “surgical site infection*”:ti,ab,kw OR “wound infection*”:ti,ab,kw OR “postoperative infection*”:ti,ab,kw

#14 #12 OR #13

#15 #3 AND #11 AND #14

#16 spine*:ti,ab,kw OR spinal:ti,ab,kw OR laminect*:ti,ab,kw OR scoliosis:ti,ab,kw

#17 #15 NOT #16
